# Role of TCTP in Cell Biological and Disease Processes

**DOI:** 10.3390/cells10092290

**Published:** 2021-09-02

**Authors:** Ulrich-Axel Bommer, Toshiaki Kawakami

**Affiliations:** 1 School of Medicine, Faculty of Science, Medicine and Health, University of Wollongong, Northfields Avenue, Wollongong, NSW 2522, Australia; 2 Laboratory of Allergic Diseases, Center for Autoimmunity and Inflammation, La Jolla Institute for Immunology, 9420 Athena Circle, La Jolla, CA 92037, USA

Translationally controlled tumor protein (TCTP), also referred to as histamine-releasing factor (HRF) or fortilin, is a multifunctional protein, expressed in essentially all eukaryotic organisms. TCTP is involved in many basic biological processes, such as stress responses, cell division, as well as growth and development, both at the cellular and the organ level. It is therefore not surprising that the dysregulation of TCTP occurs in various disease processes, such as cardiovascular, allergic and immune disorders. TCTP’s role in cancer-promoting pathways is particularly well documented, and the protein is considered to be a potential target for the design of new anti-cancer strategies. Therefore, an understanding of the core biological functions of TCTP, the mechanisms underlying its cellular regulation, and its involvement in disease processes, is essential. With this goal in mind, together with the contribution of all the authors, we compiled the Special Issue ‘Role of TCTP in Cell Biological and Disease Processes’ in *Cells*, the articles of which comprise this book. Through the inclusion of three review articles, we aimed to provide a current overview on a wide range of aspects pertaining to this protein, and four original papers highlight some recent developments in this area.

## Involvement of TCTP in Core Biological Functions

Three of these original papers reported examples of core biological processes, in which TCTP is involved. The study by S-H Chen and co-workers [1] investigated the importance of TCTP for the development of the central nervous system in mice. This group generated a conditional knockout mouse, where TCTP was disrupted in neuronal and glial progenitor cells. These mice died at the perinatal stage, and the results indicated that TCTP is a critical protein for cell survival during early neuronal differentiation.

Maria Lucibello and colleagues studied the importance of TCTP for cell division in human breast cancer cells [2]. It was known for quite some time that TCTP can be found to be associated with the mitotic spindle [3], and that it undergoes mitotic phosphorylation by the polo-like kinase Plk-1 [4], but the involvement of this process in breast cancer was only investigated much later. In their previous paper, Lucibello and co-workers showed that phospho-TCTP levels are particularly elevated in aggressive breast cancer [5]. Here, they demonstrate that phospho-TCTP is essential for correct transition through mitosis in human mammary epithelial cells.

Autophagy, a lysosomal degradation pathway, is another core biological process, which is important for maintaining homeostasis at the cellular, tissue and organism level. Only a few papers on TCTP and autophagy have been published so far, and the regulatory role of the protein in autophagy is still controversial, since both stimulation and inhibition of autophagy by TCTP were observed. Therefore, we welcomed the review article by Kyunglim Lee and colleagues [6], which discussed these aspects of TCTP function, in relation to its role in tumorigenesis. An original study by Vojtova and Hasek [7] investigated the importance of Mmi1, the yeast orthologue of mammalian TCTP, in non-selective autophagy in the yeast *S. cerevisiae*. They found that Mmi1 negatively affects rapamycin-induced autophagy, whereas it had no effect on nitrogen starvation-induced autophagy.

**Figure 1:**
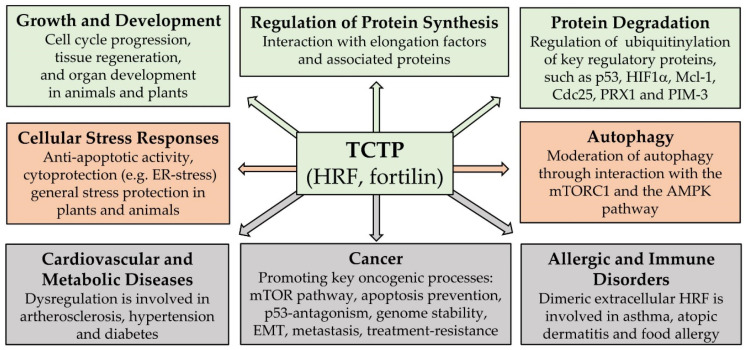
Schematic summary of core biological processes and of principal disease processes where TCTP is involved. (Green boxes: cell regulatory processes promoting growth and proliferation; orange boxes: cellular maintenance and defense systems; grey boxes: disease processes). This summary scheme is based on data compiled in the review articles [6,8,17], but also incorporates findings reported in some of the original articles of this Special Issue.

Our own review article [8] aimed to provide a comprehensive overview on the core biological functions of TCTP. Apart from the above-mentioned processes of cell/organ development and autophagy, it listed regulation of protein synthesis, stability regulation of key proteins, and biological stress responses as the main groups of cell biological processes, in which TCTP may be involved. Various interesting examples of TCTP’s activities for each of these categories can be found in this article. A general overview on the biological activities of TCTP is schematically summarized in Figure 1 (green and orange boxes). The figure is largely based on the data compiled in the review articles of this Special Issue and, apart from listing the core biological activities of TCTP, it also summarizes the main groups of disease processes, where TCTP/HRF/fortilin or its dysregulation was reported to play an important role (grey boxes). Our review article cites all the other articles of this Special Issue, where relevant, thereby putting them into context. In addition, our review provides an account of the various regulatory mechanisms that are involved in regulating cellular TCTP levels, and which are important for the understanding of some aspects of TCTP dysregulation in diseases.

## Disease Processes in Which TCTP Participates

The role of TCTP in cancer is well documented in previous reviews (for a collection of such articles, see [9]); therefore, in our review [8], we focused on the participation of TCTP in the most important cancer cell biological processes, as they are listed in the grey box in Figure 1. The above-mentioned article by Lucibello and colleagues [2] represents an original contribution to the topic ‘TCTP and cancer’. The authors addressed the question as to whether phospho-TCTP might be a suitable target for anti-cancer treatment strategies in aggressive breast cancer. They showed that treatment with dihydro-artemisinin (DHA) resulted in a reduction in phospho-TCTP levels and caused mitotic aberration in trastuzumab-resistant breast cancer cells. They also demonstrated, in an orthotopic breast cancer xenograft model that, in combinatorial treatment, DHA improved the long-term efficacy of trastuzumab emtansine (T-DM1) considerably, suggesting that targeting phospho-TCTP might be a suitable element for a combinatorial anti-cancer strategy in this type of breast cancer. The review article by Lee and colleagues [6] summarized various additional aspects of TCTP’s participation in cancer, with an emphasis on anti-apoptosis, cancer cell invasion, resistance to anti-cancer therapies, as well as the potential of TCTP as a prognostic tool in cancer.

Another group of disorders, where TCTP dysregulation was shown to be involved, are cardiovascular and metabolic diseases, such as atherosclerosis, hypertension, and diabetes (grey box in Figure 1). These observations are reviewed in our article [8] (for an earlier review also see [10], where TCTP is referred to as fortilin). An important general aspect here is that, on one hand, TCTP is important for the survival of specialized cell types, such as pancreatic β cells [11] and cardiomyocytes [12]; on the other hand, excess TCTP may lead to hypertrophy, as shown for nephrotic podocytes in diabetes [13] or in skeletal muscle [14].

Since the discovery of the histamine-releasing factor (HRF) and its molecular identification as an extracellular form of TCTP [15], it is known that TCTP/HRF is also involved in allergic and immune disorders. Unravelling the precise role of the (dimeric) extracellular form of HRF in these processes took considerable research effort, spread over more than 20 years [16]. The interesting recent developments in this area are summarized in the review article by Kawakami et al. [17] (also see the grey box in Figure 1). Another example of an extracellular activity of TCTP/HRF was revealed by the discovery that the protein is a component present in the venom of the brown spider *Loxosceles intermedia* [18]. In an effort to characterize the importance of TCTP/HRF in the spider’s venom, Senff-Ribeiro and co-workers studied the pro-inflammatory activity of TCTP in Loxoscelism, which is the reaction that develops after a bite by this spider [19]. They concluded that TCTP is an essential synergistic factor for the dermo-necrotic actions of the main toxin contained in the venom.

The extracellular function of TCTP as a histamine releasing factor, in the context of allergic reactions, was originally discovered by Susan MacDonald [15]. She and her team made a considerable contribution to elucidating several aspects related to the activity of HRF. Sadly, we learned that in September 2020, Dr. MacDonald passed away. Given her original discovery of the HRF and her further contribution to the field, we considered it appropriate to include an obituary for her in this Special Issue on TCTP/HRF [20].

## Recent Developments

Since the completion of our Special Issue, a few interesting biological activities of TCTP have been reported in non-vertebrate organisms. Some examples are its role in the growth, development and differentiation of the slime mold *Dictyostelium discoideum* [21]; the importance in *Drosophila* for epithelial integrity and organ growth [22]; and in *Toxoplasma gondii* for robust growth of the parasite and for the maintenance of its virulence [23]. Recognizing the importance of TCTP for the survival of unicellular parasites, Bossard et al. [24] took the step to test the efficacy of immunizing mice against the parasitic TCTP of *Trypanosoma brucei*. The immunized mice displayed a reduced first peak of parasitemia, a two-fold delay in the onset of the second peak and an increased time of survival, compared to the control animals.

The group of Kyunglim Lee investigated the metabolic importance of TCTP in mice. They generated a TCTP-overexpressing mouse and found that these display an improved metabolic homeostasis, with enhanced glucose tolerance, insulin sensitivity, and energy expenditure, compared to control mice [25]. In another paper, the group studied the importance of TCTP in rheumatoid arthritis (RA) [26]. They found that TCTP levels are increased in the sera and synovial fluids of patients with RA, compared to control groups. Their results indicate that TCTP might serve as a biomarker and a therapeutic target in RA patients. A novel tumor–promoting role of TCTP has just been published in Nature Immunology [27]. The authors observed that the release of TCTP from necrotic cancer cells switches on an immunosuppressive network of myeloid-derived suppressor cells, which substantially contributes to the suppression of antitumor immunity, and thus to tumor progression. All these examples show that we can still expect new facets of TCTP’s role to be unraveled, both regarding its biological and its disease-promoting activities.
